# An Assesment of The Long-Term Effects of Electroconvulsive Therapy On Cognitive Functioning İn Patients With Schizophrenia

**DOI:** 10.1192/j.eurpsy.2025.2196

**Published:** 2025-08-26

**Authors:** B. Alpuğan, D. İpekçioğlu

**Affiliations:** 1 Republic of Türkiye Ministry of Health Bitlis Tatvan Public Hospital, Bitlis; 2 Psychiatry, Istanbul Bakırköy Mazhar Osman Mental Health and Neurological Diseases Training and Research Hospital, Istanbul, Türkiye

## Abstract

**Introduction:**

In schizophrenia, cognitive symptoms emerge in the early period and are among the core symptoms. This study aimed to investigate the long-term effect of electroconvulsive therapy on the cognitive functions of schizophrenia patients.

**Objectives:**

In schizophrenia, cognitive symptoms emerge in the early period and are among the core symptoms. This study aimed to investigate the long-term effect of electroconvulsive therapy on the cognitive functions of schizophrenia patients.

**Methods:**

In this study, 25 patients diagnosed with schizophrenia according to DSM-5 criteria and treated with only pharmacotherapy (FT), 25 patients treated with pharmacotherapy plus electroconvulsive therapy (ECT) those who are inpatients in the psychiatry clinics of Istanbul Bakırköy Prof. Dr. Mazhar Osman Mental Health and Neurological Diseases Training and Research Hospital 28 healthy controls were included. Patients were evaluated clinically with tests during the acute exacerbation period and 3 months later.

**Results:**

During the acute exacerbation period, schizophrenia patients were identified to present poor cognitive performance compared to healthy controls. After three months of treatment, significant clinical improvement was observed in both patient groups. MoCA total scores increased for both groups after treatment. After treatment, TMT-A and TMT-B performance improved in the pharmacotherapy group and TMT-A performance improved in the pharmacotherapy + ECT group. With treatment, there was a significant positive change in the number of categories completed in the WCST in the pharmacotherapy group. In the Stroop Test, the pharmacotherapy group showed significant positive changes in the duration values of all cards and in the interference effect, while the pharmacotherapy + ECT group showed significant changes in the duration values of Stroop 1, 2, 4 and 5 and in the interference effect. In the pharmacotherapy + ECT group, there was a statistically significant positive correlation between the change in PANNS negative subscale scores and the duration of TMT-B and the number of completed categories, perseverative responses and perseverative errors in WCST.

**Conclusions:**

It was observed that treatment modalities are not superior to each other on cognitive functioning in the long term. The improvement in cognitive areas with treatment may be due to a decrease in symptom severity and increased patient compliance with treatment. In this field, prospective, multicenter studies with larger sample sizes, including different drug groups and different ECT modalities are needed.

**Disclosure of Interest:**

None Declared

